# Seeing in my way or your way: impact of intelligence, attention, and empathy on brain reactivity

**DOI:** 10.3389/fnhum.2023.1071676

**Published:** 2023-05-10

**Authors:** Marie-Louise Montandon, Cristelle Rodriguez, François R. Herrmann, Ariel Eytan, Alan J. Pegna, Sven Haller, Panteleimon Giannakopoulos

**Affiliations:** ^1^Department of Psychiatry, Faculty of Medicine, University of Geneva, Geneva, Switzerland; ^2^Department of Rehabilitation and Geriatrics, Geneva University Hospitals, University of Geneva, Geneva, Switzerland; ^3^Division of Institutional Measures, Medical Direction, Geneva University Hospitals, Geneva, Switzerland; ^4^School of Psychology, University of Queensland, Brisbane, QLD, Australia; ^5^CIMC—Centre d’Imagerie Médicale de Cornavin, Geneva, Switzerland; ^6^Department of Surgical Sciences, Radiology, Uppsala University, Uppsala, Sweden; ^7^Faculty of Medicine, University of Geneva, Geneva, Switzerland; ^8^Department of Radiology, Beijing Tiantan Hospital, Capital Medical University, Beijing, China

**Keywords:** automatic perspective taking, theory of mind, social cognition, fluid intelligence, fMRI

## Abstract

Previous studies showed that neurotypical adults are able to engage in unconscious analyses of others’ mental states in the context of automatic perspective taking and experience systematic difficulties when judging the conflicts between their own (Self) and another’s (Other) perspective. Several functional MRI (fMRI) studies reported widespread activation of mentalizing, salience, and executive networks when adopting the Other compared to Self perspective. This study aims to explore whether cognitive and emotional parameters impact on brain reactivity in dot perspective task (dPT). We provide here an fMRI analysis based on individual z-scores in eighty-two healthy adults who underwent the Samson’s dPT after detailed assessment of fluid intelligence, attention, levels of alexithymia and social cognition abilities. Univariate regression models were used to explore the association between brain activation patterns and psychological variables. There was a strong positive association between Wechsler Adult Intelligence Scale (WAIS) and fMRI z-scores in Self perspective. When the Other perspective is taken, Continuous Performance Test (CPT)-II parameters were negatively associated with fMRI z-scores. Individuals with higher Toronto Alexithymia scale (TAS) score and lower scores in mini-Social cognition and Emotional Assessment (SEA) displayed significantly higher egocentric interference-related fMRI z-scores. Our data demonstrate that brain activation when focusing on our own perspective depends on the levels of fluid intelligence. Decreased attentional recruitment and decreased inhibitory control affects the brain efforts to adopt the Other perspective. Egocentric interference-associated brain fMRI activation was less marked in cases with better empathy abilities but the opposite was true for persons who experience increased difficulties in the recognition of emotions.

## 1. Introduction

Empathy refers to a complex construct that plays a key role in social adaption and quality of human relationships. It includes the capacity of sharing emotions and responds immediately to the emotions of others (affective empathy) and the ability to understand what other people are thinking, imputing desires, and intentions to oneself and others (cognitive empathy) close to the concept of the theory of mind (ToM) (Decety and Jackson, 2004; Decety and Moriguchi, 2007; Young et al., 2007; Blair, 2008; de Waal, 2008). Several studies showed that ToM deficits in a variety of psychiatric disorders including autism, schizophrenia, bipolar disorder, and attention-deficit/hyperactivity disorder (Corcoran et al., 1995; Kerr et al., 2003; Abikoff et al., 2004; Blair, 2005; Bora and Pantelis, 2016; Maoz et al., 2019). There is a wide agreement that adult humans are able to engage in unconscious analyses of others’ mental states in the context of automatic perspective taking (Samson et al., 2010; Low and Watts, 2013; Surtees et al., 2016; Schneider et al., 2017). This human ability may be of key importance for the mostly unconscious ascription of mental states needed for social interactions such as cooperating with colleagues and family members, thinking about others in their absence and anticipating their emotional reactions. The psychological mechanisms surrounding this ability remains, however, matter of intense debate. Recent lines of evidence suggested the idea that ToM is controlled by two distinct systems: one explicit that deliberately considers other’s thoughts and emotions and one implicit based on the automatic analysis of their viewpoints even when such analysis is irrelevant for task processing (Onishi and Baillargeon, 2005; Surian et al., 2007; Kovacs et al., 2010; Schneider et al., 2012b). Supporting this viewpoint, infants in the second year of life appear to represent others’ false beliefs when tested using implicit looking time measures (Onishi and Baillargeon, 2005; Kovacs et al., 2010), despite their poor performances on false belief tests that require explicit, verbal responses until 4 years of age (Wellman et al., 2001). In the same line, patients with autism spectrum disorder show less evidence of implicit mentalizing than neurotypical individuals, although both neurotypical and neuro-atypical individuals perform similarly on explicit verbal ToM tasks (Senju et al., 2009; Schneider et al., 2013). Other researchers argued against this distinction and proposed that domain-general attention cueing and executive functions may explain automatic perspective taking (APT) without specificity for social stimuli (for review see Cole and Millett, 2019; Westra et al., 2021).

Neurotypical adults are prone to systematic difficulties arising from conflict between their own (self) and another’s (other) perspective. Some observations suggested that adult participants show “egocentrism” across a wide range of tasks in that their judgment of what someone else sees, are slower or more error prone when this differs from what participants themselves see (Qureshi et al., 2020). This gives rise to the widely reported phenomenon of “egocentric bias” toward the participant’s own perspective, which is almost universally observed in studies of ToM (Royzman et al., 2003; Wellman, 2014). Much of the debate regarding implicit mentalizing concerned the experimental results on the dot perspective-taking task (dPT), originally developed by Samson et al. (2010). In this task, participants are asked to count the number of dots on a screen. Importantly, an avatar is also present on the screen when the dots are revealed and sees a number of dots that is either the same as (consistent trials) or less than the number of dots that the participant sees (inconsistent trials). As expected and consistent with the notion of egocentric interference, participants take longer to report the number of dots the avatar sees when the number of dots the participant sees is different. However, participants also take longer to report how many dots they themselves see when the avatar sees a different number of dots. This second form of error was characterized as altercentric interference: participants’ ability to report their own perspective is affected by the perspective of the avatar (Qureshi et al., 2010; Samson et al., 2010). Despite the uncertainties about the specificity of the psychological mechanisms surrounding egocentric and altercentric interference, there is no doubt that these parameters may impact on social interactions both in general population and clinical samples (Furlanetto et al., 2016; Drayton et al., 2018; Gao et al., 2019; Del Sette et al., 2022). As an example, compared to younger persons in dPT, individuals older that 55 years show an increase sensitivity to other’s conflicting viewpoint as documented by increased reaction time in case of incongruence when adopting their own perspective (Martin et al., 2019).

The brain substrates of dPT and in particular egocentric and altercentric interference are complex and remain matter of intense debate. Early functional MRI (fMRI) observations showed an activation of the dorsolateral prefrontal, parietal cortices when selecting one’s own over another’s visual perspective, and vice versa mainly in case of conflict between the two perspectives (Ramsey et al., 2013). In the same line, a domain-specific activation in several cortical areas such as right temporo-parietal junction and ventral medial prefrontal cortex was described in inconsistent trials when selecting one’s own perspective (Schurz et al., 2015), although this position has been also challenged (Santiesteban et al., 2017). The right temporo-parietal junction stimulation significantly improved the judgment from the allocentric perspective, whereas stimulation of the dorsomedial prefrontal cortex improved performance from the egocentric perspective (Yao et al., 2021). Using transcranial direct current stimulation during a visual perspective task, (Martin et al., 2017) also reported a key role of dorsomedial prefrontal cortex in the integration of external information when adopting the self-perspective. In a meta-analysis of fMRI findings, (Arora et al., 2017) stressed the paucity of fMRI studies focusing on dPT and reported no consistent overlap of dPT activation patterns with the ToM core regions.

Besides limited samples and absence of concomitant consideration of Self versus Other and Consistent versus Inconsistent effect on brain activation, one main limitation of these studies is that they did not take into account cognitive and emotional parameters that, at an individual level, impact on the expression of APT in humans such as attention processing, inhibitory control, but also social cognition (Schneider et al., 2012a; Burnside et al., 2017; Pineda-Alhucema et al., 2018). More recently, it has been reported that people with lower attentional resources and decreased inhibitory control [measured with the Conner’s Continuous Performance Test-II (CPT-II)] display worse performances in the dPT task and make more errors when judging conflicting perspectives both according to their own and other viewpoints (Rodriguez et al., 2022). However, this latter study concerned only clinical observations without reference to dPT-related brain activation patterns. The present report aims to explore whether psychological variables characteristic of each individual determine the brain reactivity in dPT. Our hypothesis was that the level of fluid intelligence, regulation of attentional resources and inhibitory control but also identification and expression of emotions as well as ToM global performance might impact on the activation of neural generators needed to deal with conflicting views when judging our own versus other’s perspective. This fMRI study explores differences in individual z-scores (rather than group differences in patterns of brain activation) in eighty-two healthy adults who underwent a detailed assessment of fluid intelligence, attention, levels of alexithymia and social cognition abilities prior to the administration of the Samson’s dPT. Taking into account the previous contributions in this field (Schneider et al., 2012a; Kanske et al., 2015; Burnside et al., 2017; Pineda-Alhucema et al., 2018; Rodriguez et al., 2022) we expected that deficits in attention and inhibitory control would be associated with increased egocentric and altercentric interference-related BOLD signal. We also postulated that decreased abilities in the recognition of emotions and ToM would increase brain reactivity to egocentric interference.

## 2. Materials and methods

### 2.1. Participants

The study was approved by the local Ethics Committee and all participants gave written informed consent prior to inclusion. All the cases were recruited via advertisements in local newspapers and media. The present sample included 82 community-dwelling men (mean age 32.7 ± 11.4 years, range age 19−66 years). Subjects with presence of history of a chronic psychiatric disorder (psychosis or bipolar disorder), history of loss of consciousness lasting longer than 30 min, history of head injury or post-concussion symptoms, history of auditory or visual deficits, seizure and neurological disorders, and regular use of psychotropic medications were excluded. Structural brain abnormalities were excluded after routine radiological assessment. This work was based on healthy controls who accepted to participate in the fMRI activation study. The final sample was formed by both cases included in our previous study that focused on the cognitive and emotional determinants of dPT (Rodriguez et al., 2022) and independent sample of newly recruited cases.

### 2.2. Psychological assessment

According to our *a priori* hypotheses, this assessment concerned fluid intelligence, attention and inhibitory control, emotion perception, and social cognition.

The Wechsler Adult Intelligence Scale (WAIS) is a general intelligence battery (Wechsler, 2011) used to evaluate patient’s intelligence quotient (IQ). The ten core subtests of the battery give rise to four index scores including the Verbal Comprehension Index, the Perceptual Reasoning Index, the Working Memory Index, and the Processing Speed Index.

The CPT-II is a computerized measure of inattentiveness, inhibitory control, sustained attention, and vigilance (Conners, 2004). As such, letters are presented on the screen one at a time at interstimulus intervals of 1, 2, and 4 s, with a presentation time of 250 ms. The participant is instructed to respond by clicking the space bar to every letter except for the “X” as quickly as possible. CPT-II outcome variables include hit reaction time to correct responses (HRT), standard error of HRT (HRT SE), omission errors (missed targets), commission errors (incorrect responses to non-targets), and detectability (ability to discriminate between targets and non-targets).

The mini-Social cognition and Emotional Assessment (SEA) is a quick clinical tool that assesses ToM and emotion recognition deficits. One part is a reduced and modified version of the faux-pas test, and the second part is a reduced version of Paul Ekman emotion recognition test (Bertoux et al., 2014), resulting in two computational scores (ToM and emotion recognition) and a general composite score. In the shorted faux-pas test, ten stories are presented, including five social faux-pas stories and 5 control stories without any faux-pas. Participants are asked to detect and explain faux-pas as well as to make interferences about intentions, beliefs and feelings of other’s (Was a faux-pas present? What was the faux-pas? Who made it? Why? Was it intentional? How did the victim feel?). In the reduced (35 faces) emotion recognition test, participants identify which emotion was expressed among a list of seven different emotions (fear, sadness, disgust, surprise, anger, happiness, and neutral) depicted in a series of photographs. The two subtests of the mini-SEA result in two computational scores (ToM and emotion recognition) converted to the composite subscores (from 0 to 15, respectively), and in an overall mini-SEA composite score (maximum score of 30) obtained by adding the two composite subscores.

The Geneva Social Cognition Scale (GeSoCS) is a medium duration assessment tool that detects and characterizes significant changes in social cognition and ToM. It is a 100-point scale composed of 6 subtests: ToM stories, recognition of social emotions, false beliefs, inferences, absurdity judgment, and planning abilities (Martory et al., 2015).

The French version of the Toronto Alexithymia Scale [TAS (Pinaquy et al., 2002)] is a 20-item instrument that is one of the most commonly used measures of alexithymia. Alexithymia refers to people who have trouble identifying and describing emotions and who tend to minimize emotional experience and focus attention externally. Items are rated using a five-point Likert scale whereby 1 = strongly disagree and 5 = strongly agree. There are 5 items that are negatively keyed (items 4, 5, 10, 18, and 19). The total alexithymia score is the sum of responses to all 20 items. The TAS-20 uses cutoff scoring: equal to or less than 51 = non-alexithymia, equal to or greater than 61 = alexithymia. Scores of 52−60 = possible alexithymia. Research has yielded considerable evidence that the French version of TAS-20 is a reliable and valid measure of alexithymia in normal and clinical adult and adolescent samples. In adults, the value of the Cronbach alpha was 0.79 and the correlation between each item and the total score ranges from 0.19 (*p* < 0.05) to 0.69 (*p* < 0.001) with a mean of 0.52 (Loas et al., 1995).

### 2.3. Dot perspective taking task

**FIGURE 1:**
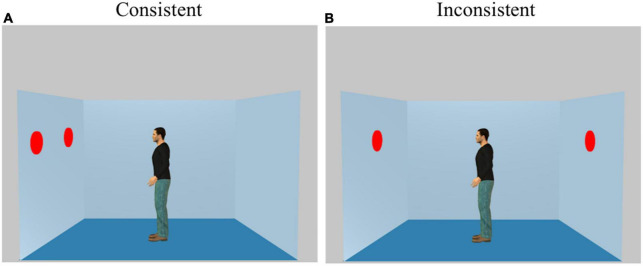
Examples of the stimuli presented in **(A)** Consistent and **(B)** Inconsistent conditions. Each trial included four stimuli, presented in the center of the screen in the following order: (i) a fixation cross indicating the start of the trial, (ii) a word indicating whether participants should adopt their own perspective (“YOU”) or the perspective of the avatar (“HE”), (iii) a number of discs (0–3) specifying the content to be verified, and (iv) a picture of the avatar in a room. Stimuli i–iii each appeared for 750 ms, and each was followed by a blank screen for 500 ms. After the final stimulus, participants had 2,000 ms to adopt the perspective indicated and judge whether the number of discs was the same (“yes” response), or not (“no” response). The next trial was delivered after 2,000 ms if no response was given. Participants did not receive any trial-by-trial feedback about their performance. Trials lasted 5,750 ms each and were separated by 6,500 ms rest periods.

We used an adapted computer-based response-time task developed by Samson et al. (2010) (Experiment 1). The stimuli consisted of a picture showing a lateral view into a room with the left, back, and right walls visible. Red discs were displayed on one or two walls. A human avatar always appeared in the center of the room in profile facing either the right or the left wall. Depending upon the orientation of the avatar and the positioning of the discs, the avatar was able or unable to see all the discs in the room. On each trial, participants judged either their own visual perspective (Self trials) or the visual perspective of the avatar (Other trials) (Figure 1). Specifically, participants were asked to verify the number of discs that either they (Self) or the avatar (Other) could see. On 50% trials, the participant and the avatar could see the same number of discs (Consistent perspective condition). On 50% trials, they could see a different numbers of discs (Inconsistent perspective condition). The position of the avatar was kept constant across consistent and inconsistent trials, but the position of the discs changed. Each trial included four stimuli, presented in the center of the screen in the following order: (i) a fixation cross indicating the start of the trial, (ii) a word indicating whether participants should adopt their own perspective (“YOU”) or the perspective of the avatar (“HE”), (iii) a number of discs (0−3) to be verified, and (iv) a picture of the avatar in a room. Stimuli i−iii each appeared for 750 ms, and each was followed by a blank screen for 500 ms. After the final stimulus, participants had 2,000 ms to adopt the perspective indicated and judge whether the number of discs was the same (“yes” response), or not (“no” response). The next trial was delivered after 2,000 ms if no response was given. Participants did not receive any trial-by-trial feedback about their performance. Trials lasted 5,750 ms each and were separated by 6,500 ms rest periods. The whole trials were analyzed and the rest periods were considered as control events. Trials were presented in four blocks, each consisting of 36 trials. Each block also included 4 filler trials in which there were no discs on the walls of the room. When Self perspective was selected (“YOU”), participants should answer “0” to the question about the number of discs, and “yes” to whether the subject had the same perspective as the avatar. The order of presentation of the blocks was randomized and counterbalanced across participants. The entire procedure was conducted using E-Prime 3.0 software to control the stimulus presentation and data collection.^1^

Anticipatory responses (<200 ms) or delayed responses (>2,000 ms) were counted as errors. The response times were log-transformed to normalize their distribution. Percentage of errors and reaction times were assessed for each of the four trial types (Self Consistent, Self Inconsistent, Other Consistent, and Other Inconsistent). Altercentric and egocentric interference RT correspond to the subtraction of two conditions (Self Consistent-Self Inconsistent, Other Consistent-Other Inconsistent). Erroneous responses in all of the conditions (Self, Other, Consistent, Inconsistent) were considered to define the percentage of errors for each participant.

### 2.4. MR imaging

MR imaging were acquired using a 3T MRI scanner (MAGNETOM Prisma, Siemens Healthineers, Erlangen, Germany) at Campus Biotech Geneva.^2^ Functional echo-planar imaging had the following essential parameters: 66 slices, slice thickness = 2.0 mm, voxel size = 2.0 × 2.0 × 2.0 mm^3^, repetition time = 1,000 ms, echo time = 32 ms, flip angle = 50°, field of view = 224 mm, resulting in 8.05 min per fMRI run. Each participant performed the 4 runs in a pseudo-randomized design. An additionally acquired 3DT1 sequence (208 slices; slice thickness = 1.0 mm; voxel size = 1 × 1 × 1 mm^3^; repetition time = 2,300 ms; echo time = 2.26 ms; flip angle = 8°; field of view = 256 mm) was used for spatial normalization and registration.

## 3. Statistical analysis

### 3.1. GLM analyses of the task-related activation

Task-related general linear model (GLM) data processing was carried out using FEAT (FMRI Expert Analysis Tool) Version 6.0.2, part of FSL (FMRIB’s Software Library).^3^ At the first level, we performed a within-session analysis. At the second level, we input the data from Level 1 and estimated each participant’s mean response. At the third level, the group across all 82 participants was calculated. Higher-level analysis was carried out using a mixed effects model, by forcing the random effects variance to zero in FLAME (FMRIB’s Local Analysis of Mixed Effects) (Beckmann et al., 2003; Woolrich et al., 2004; Woolrich, 2008). Z (Gaussianised T/F) statistic images were thresholded using clusters determined by *Z* > 3.1 and a corrected cluster significance threshold of *P* = 0.05. Anatomic location of the activation clusters was determined using “atlasquery,” part of FSL, and the Harvard–Oxford Cortical Structural Atlas. The thresholded z-score maps were determined for the main effect of Self and Other conditions, Consistent and Inconsistent situations, as well for the contrasts Self Inconsistent versus Self Consistent and Other Inconsistent versus Other Consistent. Then, those z-score maps were applied to the corresponding individual maps of each participant resulting in individual z-scores. For example, the individual z-score of participant 1 for Self condition is the average individual z-scores of all individual voxels of participant 1 in condition Self using as a mask the group average result for the condition Self.

### 3.2. Regression models

Paired *t*-test were used to compare the average reaction time and percentage of errors between different trial types and conditions. Degree of freedom for paired *t*-test are computed as N-1. Linear regression models were used to explore the aged adjusted association between individual fMRI z-scores and WAIS global score (as well as the score of subscales), CPT-II parameters, TAS, and mini-SEA scores (independent variables). In linear regression, the *t*-test for an estimator has N–P–1 degrees of freedom (dof) where N is the number of observation and P number of explanatory parameters in the model.

Correction for multiple analysis was made using the Benjamini–Hochberg method. We applied the Benjamini–Hochberg correction in three different sets of variables as a function of *a priori* hypotheses: general intelligence (WAIS), attentional resources (CPT-II) and markers of emotional identification and expression (TAS-20) and social cognition (mini-SEA). The corrected *p*-values took into account all of the comparisons made for each set of variables in Self and Other conditions as well as Inconsistent and Consistent conditions (Green and Diggle, 2007). Effect size was estimated with Eta^2^, a measure of the proportion of variance associated with each variable. A correlation matrix was built with Pearson correlation coefficient (r) and potential multicollinearity among the psychological tests was tested using the variance inflation factor (VIF) as an indicator. Degree of freedom for the Pearson correlation coefficient are computed as N-2.

## 4. Results

### 4.1. Behavioral data

**FIGURE 2:**
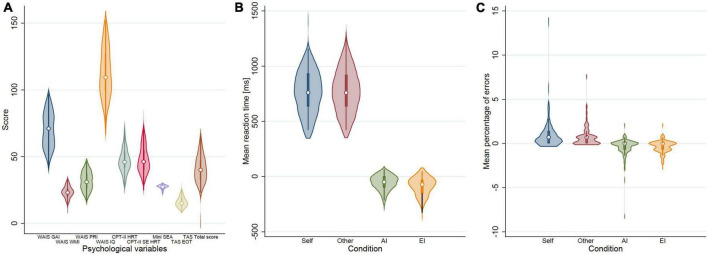
Violin plots showing **(A)** the distribution of psychological variables. WAIS: general ability index (GAI), Working Memory Index (WMI), Perceptual Reasoning Index (PRI), IQ Total score; CPT-II: Hit reaction time (HRT), Standard error of consistency of HRT (SE HRT); mini-SEA; TAS: External oriented thought (EOT), Total score; **(B)** the distribution of reaction time by conditions of interest; **(C)** mean percentage of errors by conditions of interest. Altercentric and egocentric interference RT correspond to the subtraction of two conditions (Self Consistent-Self Inconsistent, Other Consistent-Other Inconsistent). Average RT differences are negative since participants need more time to deal with inconsistent trials.

The distribution of psychological variables as well as mean reaction times in dPT are displayed in Figure 2. Mean reaction times and percentage of errors for Self and Other and Consistent and Inconsistent conditions are summarized in Table 1. Mean reaction times were similar for Self and Other conditions. However, there was a marked increase in mean reaction times in Self Inconsistent compared to Self Consistent (*t* = −7.45, *P* < 0.0001) and in Other Inconsistent compared to Other Consistent (*t* = −9.4, *P* < 0.0001) conditions (Table 1). The Self-Other comparison yielded a significant increase of the percentage of errors in Self condition (*t* = 3.45, *P* < 0.001). As for mean reaction times, inconsistency was associated with a significant increase of the percentage of errors both in Self and Other conditions (*t* = −3.6, −4.7, *P* < 0.005, and *P* < 0.0001, respectively) (Table 1). Degree of freedom all paired *t*-test is 81. Psychological tests were at most moderately correlated (mini-SEA and total IQ score: *r* = 0.47, *p* < 0.001; GeSoCS and total IQ score: *r* = 0.34, *p* < 0.001). Multicollinearity among psychological tests was not an issue as VIF was consistently lower than 10 (with a maximum observed value of 1.44 for the total IQ score). Degree of freedom for the Pearson correlation coefficient is 80.

**TABLE 1:** Participants’ characteristics (*N* = 82).

Variable	Mean ± SD, 95% CI or N (%)
Age	33.1 ± 10.4, 19.0–66.0
**Education**	
<12 years	0 (0.0%)
12 years	21 (25.6%)
>12 years	61 (74.4%)
Right-handedness	77 (93.9%)
**Neuropsychological tests**	
WAIS	
Verbal comprehension index	38.2 ± 8.1, 23.0–54.0
Perceptual reasoning index	31.7 ± 7.2, 16.0–46.0
Working memory index	23.1 ± 4.9, 9.0–34.0
Processing speed index	19.9 ± 4.2, 9.0–33.0
IQ total score	112.9 ± 19.2, 67.0–152.0
GAI	69.9 ± 13.6, 42.0–97.0
CPT-II	
Omission errors	50.2 ± 13.9, 39.9–122.9
Commission errors	49.8 ± 9.4, 32.9–74.0
Detectability	48.9 ± 9.1, 23.4–65.0
Hit reaction time (HRT)	47.0 ± 9.6, 25.5–73.5
Standard error of HRT	48.2 ± 10.7, 25.0–81.1
Mini-SEA total score	27.0 ± 2.0, 20.6–30.0
Faux-pas test	14.2 ± 1.5, 8.6–15.0
Emotion recognition test	12.8 ± 1.1, 9.4–15.0
GeSoCS total score	90.4 ± 5.6, 72.0–98.0
TAS Total score	40.7 ± 10.8, 0.0–67.0
External oriented thought	16.2 ± 4.3, 9.0–29.0
**Visual perspective-taking task**	
Mean reaction time (ms)	
Self-condition (ms)	778.96 ± 213.18, 732.1 825.8
Other condition (ms)	778.68 ± 198.81, 735.0 822.4
Altercentric interference (self) (ms)	−59.65 ± 72.66, −75.6 −43.7
Egocentric interference (other) (ms)	−91.85 ± 88.68, −111.3 −72.4
Percentage of errors	
Self	1.0 ± 1.9, 0.0−13.9
Other	0.8 ± 1.2, 0.0−7.6
Altercentric interference (Self)	−0.5 ± 1.2, −8.3 to 2.1
Egocentric interference (Other)	−0.4 ± 0.7, −2.8 to 2.1
Individual fMRI z-scores in dPT	
Self	201.2 ± 123.3, −63.9 to 485.3
Other	204.7 ± 146.1, −171.7 to 542.6
Altercentric interference (Self)	17.0 ± 31.9, −69.4 to 117.1
xEgocentric interference (Other)	32.6 ± 45.4, −82.2 to 141.6

### 4.2. fMRI activation patterns

**FIGURE 3:**
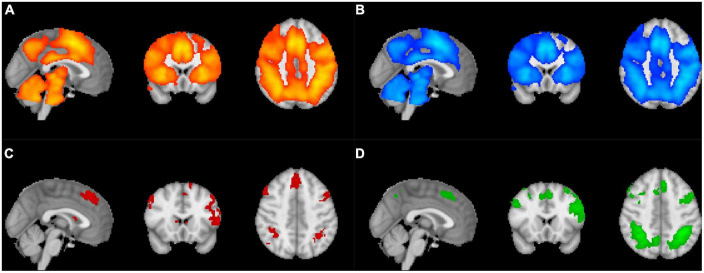
Illustration of the thresholded z-score maps applied to the corresponding individual maps of each participant resulting in individual z-scores. The thresholded z-score maps were determined for the main effect of **(A)** Self, **(B)** Other, **(C)** Self Inconsistent versus Self Consistent, and **(D)** Other Inconsistent versus Other Consistent conditions.

In the task-related GLM analysis, the comparison “Self versus Other” revealed strong BOLD activation in the left lingual and supramarginal gyri. Additional activations were present in the occipital cortex, notably the primary visual area, related to the visual stimulus presentation. The inverse comparison generated higher activation in the right lateral occipital and precuneus cortices, the left fusiform cortex, the left medial and orbitofrontal cortex, as well as the posterior cingulate gyrus. The contrast “Self Inconsistent versus Self Consistent” showed and activation of the lateral occipital cortex bilaterally, the right supramarginal gyrus and the bilateral angular gyrus, and the bilateral inferior, superior and middle frontal gyri. The average activation of the contrast “Other Inconsistent versus Other Consistent” yielded strong activation in the bilateral lateral occipital cortex, precuneus cortex and superior parietal lobule, and in the middle, superior, precentral gyri, and left frontal pole (Figure 3).

### 4.3. Association between clinical variables and individual fMRI z-scores in dPT

**FIGURE 4:**
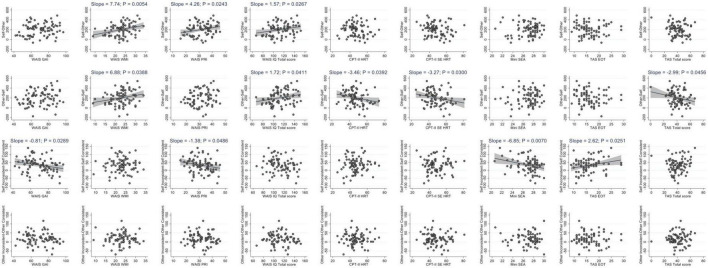
Scatterplots illustrating the univariate association between clinical variables and z-scores according to the fMRI contrasts of interest (Self-Other, Other-Self, Self Inconsistent-Self Consistent, Other Inconsistent-Other Consistent). Regression line are shown when the slope is significantly different from zero.

Regression models adjusted for age were used to explore the association between brain activation patterns in dPT and psychological measures in the present series. There was a strong positive association between WAIS working memory index, perceptual reasoning index as well as total IQ score and individual fMRI z-scores in Self-Other contrast (activation of Self perspective). Of importance, this association persisted when correcting for multiple comparisons using an adapted Benjamini–Hochberg *p* threshold. When the effect size was concomitantly considered, the WAIS working memory index was the best predictor of Self-Other contrast fMRI z-scores. CPT-II, mini-SEA, and TAS scores were not associated with fMRI activation in Self-Other contrast (Table 2; Figure 4).

**TABLE 2:** Association of neuropsychological tests (independent variables) with fMRI z-scores in dPT (self and other conditions) using age-adjusted linear regression.

Condition	Neuropsytests	Coeff. (95% CI)	*P*	P_BH_	Eta^2^ (95% CI)
**Self-other**					
	WAIS				
	GAI	1.67 (−0.40, 3.74)	0.113	0.038	0.032 (0.136)
	Working memory index	7.66 (2.23, 13.10)	0.006*	0.038*	0.091 (0.008,0.222)*
	Perceptual reasoning index	4.30 (0.43, 8.16)	0.030*	0.038*	0.058 (0.179)
	IQ total score	1.57 (0.13, 3.00)	0.033*	0.038*	0.056 (0.176)
	CPT-II				
	Hit reaction time (HRT)	−1.82 (−4.80, 1.17)	0.230	0.025	0.018 (0.111)
	Standard error of consistency of HRT	−2.33 (−4.89, 0.22)	0.073	0.025	0.040 (0.151)
	Mini-SEA	2.26 (−11.79, 16.31)	0.750	0.017	0.001 (0.056)
	TAS				
	External oriented thought	−1.93 (−8.24, 4.37)	0.544	0.017	0.005 (0.076)
	Total score	−2.04 (−4.55, 0.47)	0.109	0.017	0.032 (0.137)
**Other-self**					
	WAIS				
	GAI	1.93 (−0.52, 4.39)	0.121	0.013	0.030 (0.134)
	Working memory index	6.82 (0.23, 13.41)	0.043*	0.013	0.051 (0.168)
	Perceptual reasoning index	4.12 (−0.51, 8.75)	0.081	0.013	0.038 (0.147)
	IQ total score	1.73 (0.03, 3.44)	0.047*	0.013	0.049 (0.165)
	CPT-II				
	Hit reaction time (HRT)	−4.08 (−7.54, −0.63)	0.021*	0.050*	0.065 (0.001,0.189)*
	Standard error of consistency of HRT	−3.49 (−6.49, −0.49)	0.023*	0.050*	0.064 (0.000,0.186)*
	Mini-SEA	7.96 (−8.63, 24.54)	0.343	0.017	0.011 (0.095)
	TAS				
	External oriented thought	−3.37 (−10.93, 4.18)	0.376	0.017	0.010 (0.092)
	Total score	−2.99 (−5.94, −0.04)	0.047*	0.017	0.049 (0.165)

*P_BH_*: *p*-value according to Benjamini–Hochberg correction for multiple comparisons. *N* = 82, *P* = 2, thus degree of freedom (dof) of the *t*-test for an estimator is 79, with the exception of TAS External oriented thought, where *N* = 82 and dof is 78. Effect size is expressed as Eta^2^. *Indicates significant values.

When the Other perspective is taken (Other-Self contrast), fMRI z-scores were positively related to WAIS working memory index, and total IQ score. In contrast, increased HRT and standard error of consistency of HRT in CPT-II were negatively associated with fMRI z-scores in this condition. This was also the case for the TAS total score. However, when correcting for multiple comparisons, only the CPT-II parameters remained significant predictors of Other-Self contrast fMRI z-scores. This association persisted when the effect size was considered (Table 2).

The fMRI z-scores for the Self Inconsistent-Self Consistent contrast correspond to the brain activation associated with altercentric interference (other’s viewpoint automatically affects the judgment of own’s perspective). Importantly, neither of the psychological parameters studied here affects this imaging parameter (Table 3). WAIS general ability index (GAI) and perceptual reasoning index were negatively related to brain activation related to egocentric interference (Other Inconsistent-Other Consistent contrast). This was also the case for the mini-SEA. In contrast, there was a positive association between the TAS external oriented thought and fMRI patterns of egocentric interference. After Benjamini-Hochberg correction, mini-SEA and TAS external oriented thought scores persisted as main predictors (negative and positive, respectively) of fMRI z-score differences in egocentric interference. In terms of effect size, mini-SEA scores remained the only significant predictor (negative) of fMRI z-scores for this contrast (Table 3).

**TABLE 3:** Association of neuropsychological tests (independent variables) with individual fMRI z-scores in dPT using age-adjusted linear regression.

Condition	Neuropsytests	Coeff. (95% CI)	*P*	P_BH_	Eta^2^ (95% CI)
**Self consistent-self inconsistent**					
	WAIS				
	GAI	−0.28 (−0.82, 0.27)	0.312	0.013	0.013 (0.099)
	Working memory index	−0.04 (−1.52, 1.44)	0.959	0.013	0.000 (0.012)
	Perceptual reasoning index	−0.36 (−1.39, 0.67)	0.486	0.013	0.006 (0.080)
	IQ total score	−0.22 (−0.60, 0.16)	0.261	0.013	0.016 (0.106)
	CPT-II				
	Hit reaction time (HRT)	0.05 (−0.74, 0.83)	0.908	0.025	0.000 (0.031)
	Standard error of consistency of HRT	−0.12 (−0.80, 0.56)	0.729	0.025	0.002 (0.058)
	Mini-SEA total score	−0.58 (−4.23, 3.07)	0.752	0.017	0.001 (0.055)
	TAS				
	External oriented thought	−0.22 (−1.90, 1.46)	0.793	0.017	0.001 (0.051)
	Total score	0.21 (−0.45, 0.87)	0.522	0.017	0.005 (0.077)
**Other consistent-other inconsistent**					
	WAIS				
	GAI	−0.90 (−1.65, −0.15)	0.019*	0.013	0.067 (0.001, 0.191)
	Working memory index	0.11 (−1.99, 2.22)	0.914	0.013	0.000 (0.030)
	Perceptual reasoning index	−1.55 (−2.98, −0.13)	0.033*	0.013	0.056 (0.175)
	IQ total score	−0.50 (−1.04, 0.03)	0.064	0.013	0.043 (0.155)
	CPT-II				
	Hit reaction time (HRT)	0.17 (−0.94, 1.29)	0.756	0.025	0.001 (0.055)
	Standard error of consistency of HRT	0.05 (−0.91, 1.02)	0.914	0.025	0.000 (0.030)
	Mini-SEA total score	−6.84 (−11.80, −1.89)	0.007*	0.033*	0.087 (0.006, 0.218)*
	TAS				
	External oriented thought	2.61 (0.32, 4.90)	0.026*	0.033*	0.062 (0.184)
	Total score	0.55 (−0.38, 1.48)	0.244	0.033	0.017 (0.108)

*P_BH_*: *p*-value according to Benjamini-Hochberg correction for multiple comparisons. *N* = 82, *P* = 2, thus degree of freedom (dof) of the *t*-test for an estimator is 79, with the exception of TAS External oriented thought, where *N* = 82 and dof is 78. Effect size is expressed as Eta^2^. *Indicates significant values.

## 5. Discussion

To our knowledge, this is the first study attempting to explore at an individual level the impact of cognitive and emotional parameters on the activation of human brain during automatic perspective taking. Individual fMRI z-scores when focusing on Self perspective depend on the levels of fluid intelligence. Among cognitive parameters, decreased attentional recruitment and decreased inhibitory control is related to lower fMRI z-scores when adopting the Other’s perspective. Brain activation associated with egocentric interference was significantly lower in cases with better empathy abilities but significantly higher in persons with higher levels of alexithymia (implying increased difficulties in the recognition of emotions).

The association between fluid intelligence measures and fMRI z-scores concerned only the Self condition in the present series. In other words, persons with higher levels of intelligence display increased brain activation when focusing on their own perspective. The increased recruitment of neural resources in the Self condition among persons with higher WAIS scores is consistent with several fMRI articles showing stronger brain activity (mainly in frontoparietal networks) in the presence of higher fluid intelligence in sleep, resting state and following activation with a variety of cognitive paradigms (Gray et al., 2003; Fang et al., 2019, 2022; Assem et al., 2020; Santarnecchi et al., 2021). Brain activation in our experimental setting does not depend on the levels of attention and inhibitory control (as measured by CPT-II parameters). Intriguingly, the effect of fluid intelligence on fMRI z-scores was not found when taking the Other’s perspective. In this situation, individuals with decreased attention and inhibitory control (independently of their WAIS performances) displayed lower fMRI z-scores. Earlier studies reported that attentional orienting contributes decisively to performance in the dPT (Bukowski and Samson, 2017; Gardner et al., 2018). In our recent behavioral analysis using the same experimental paradigm, it has been shown that people with decreased levels of attention and decreased inhibitory control may need more time and make more errors when judging the Other’s perspective (Rodriguez et al., 2022). Extending these observations by adding fMRI data, our results imply that, compared to the Self condition, the brain activation needed for taking the Other’s perspective is more difficult to sustain in the presence of lower attentional resources and decreased inhibitory control. Taken together, these findings reveal a dissociation between the psychological factors that may determine brain reactivity during perspective taking. The level of fluid intelligence contributes to individual differences in brain activation in “Seeing it on my way” condition. In “Seeing it on the Other’s way” condition, these latter depend, at least partly, on preserved attention and inhibitory control.

One main focus of the present study was the analysis of the determinants of brain activation associated with altercentric versus egocentric interference. The individual fMRI z-scores for the Self Inconsistent-Self Consistent contrast (altercentric interference) as well as for the Other Inconsistent- Other Consistent contrast (egocentric interference) reported here provide a fMRI correlate of these interferences. In our models, the fMRI z-scores associated with altercentric interference were not related to fluid intelligence, attention, social cognition, and levels of alexithymia. An opposite scenario is present for the egocentric interference. The fMRI z-scores for this contrast are higher in cases with worst abilities of social cognition. This observation extends earlier behavioral reports by Bukowski and Samson (2016) and Mattan et al. (2016) who reported that lower levels of empathy and negative emotions are associated with higher interference in case of conflicting perspective. Altogether these observations imply that, from an imaging standpoint, the “egocentric” bias when adopting the Other’s perspective could be more pronounced in persons less able to deal with emotional challenges and decreased abilities in theory of mind and affective empathy.

The present data suggest that neurocognitive and psychological attributes should be taken into account when interpreting the individual differences of brain activation in dPT. The fact that distinct cognitive and emotional parameters impact on fMRI z-scores related to Self and Other perspective but also altercentric and egocentric interference should be interpreted within the theoretical framework of the link between emotional regulation, cognition and ability to take other’s perspective. Most previous reports in this field concerned clinical populations. The dPT performances may be preserved in psychiatric pathologies affecting intelligence but also emotional regulation such as autism spectrum conditions and alcoholism (Pearson et al., 2013; Cox et al., 2016; Sijtsma et al., 2020). In contrast, they are known to be affected in patients with attention deficits syndrome (Nilsen et al., 2013). High levels of egocentric interference has been also reported in narcissistic personality (Bukowski and Samson, 2021) whereas lower performances in dPT were found in patients with mixed personality and anxiety disorders (Czekoova et al., 2020; Shaw et al., 2020). In the present study of healthy controls, decreased fluid intelligence, levels of attention and inhibitory control but also worst identification and expression of emotions as well as decreased social cognition abilities impact negatively on brain reactivity during dPT. To date, no fMRI data are available to explore whether these observations are valid in clinical samples. It would be thus of interest to investigate the fMRI z-scores in prodromal and mild cases with deficient inhibitory control such as obsessive-compulsive disorder (Mancini et al., 2018), primary motor stereotypies (Mirabella et al., 2020), autism spectrum disorder (Schmitt et al., 2018), Parkinson’s disease (Di Caprio et al., 2020), and attention-deficit/hyperactivity disorder (van Hulst et al., 2018).

The strengths of this report include its use of a large sample, independent variables assessing cognitive processes such as attention and fluid intelligence, general levels of empathy, but also inhibitory control and stringent control for multiple comparisons in the regression models. Some limitations should also be noted. Conceptually, one should keep in mind that APT is one among the facets involved in social cognition. For some authors, the unconscious impact of other’s divergent viewpoint when we focus on our own visual experience is mostly driven by the activation of self-other distinction, self-updating via integration of self-relevant information, central executive functions and mirroring (Bukowski, 2018; Alcala-Lopez et al., 2019). Our data did not address the correlations between these cognitive dimensions and brain reactivity. In the same line, the present findings concern only the Samson’s dPT and are not applicable to ToM paradigms involving how the objects and their arrangement may look to another person. All of our cases were socially integrated young men without history of criminal convictions and substance abuse who were initially recruited as part of a study focusing on psychopathy in the context of forensic psychiatry. The absence of women as well as the careful exclusion of neurological and psychiatric disorders as well as regular use of psychotropics, as well as scores of all of the cognitive and emotional variables within the normal range limit the generalizability of our observations. Studying dPT-related patterns of fMRI activation in persons with clinically overt learning and attention deficits as well as disorders characterized by high levels of alexithymia and decreased abilities of social cognition such as antisocial and schizoid personality (including Asperger syndrome) could be of particular interest in the current effort to define diagnostic and prognostic markers for these conditions.

## Data availability statement

The raw data supporting the conclusions of this article will be made available by the authors, without undue reservation.

## Ethics statement

The studies involving human participants were reviewed and approved by the Commission Cantonale d’Éthique de la Recherche (CCER). The patients/participants provided their written informed consent to participate in this study.

## Author contributions

PG, SH, AP, and M-LM: study conceptualization. M-LM: conceptualize the visual perspective-taking task. CR and M-LM: recruitment. M-LM, CR, and PG: neuropsychology supervising. M-LM and FH: data preparation. M-LM, FH, and PG: analyze the data. PG, FH, M-LM, and SH: manuscript writing. All authors contributed to the article and approved the submitted version.

## Footnotes

1
https://pstnet.com/products/e-prime/

2
https://www.campusbiotech.ch/

3
www.fmrib.ox.ac.uk/fsl
